# Editorial: Functional foods, supplements, and dietary approaches in sports and clinical nutrition

**DOI:** 10.3389/fnut.2023.1203477

**Published:** 2023-05-05

**Authors:** Heitor O. Santos, Scott C. Forbes, Mihnea-Alexandru Găman

**Affiliations:** ^1^School of Medicine, Federal University of Uberlandia (UFU), Uberlandia, Minas Gerais, Brazil; ^2^Faculty of Education, Department of Physical Education Studies, Brandon University, Brandon, MB, Canada; ^3^Faculty of Medicine, “Carol Davila” University of Medicine and Pharmacy, Bucharest, Romania; ^4^Department of Hematology, Center of Hematology and Bone Marrow Transplantation, Fundeni Clinical Institute, Bucharest, Romania

**Keywords:** herbal medicines, functional food, supplements, cardiometabolic risk, sports nutrition, clinical nutrition

Nutritional research includes the investigation of various dietary approaches, functional foods, and supplements, to guide personalized advice to enhance health and/or performance (1–8). Evidence-based decisions must be made based on the most recent advances in research. Therefore, we created a special issue entitled “*Functional Foods, Supplements, and Dietary Approaches in Sports and Clinical Nutrition*”, which consisted of a number of leading experts. In the special issue, 7 papers were focused on health or clinical populations and 4 manuscripts had a sport or performance focus. In this editorial, we briefly highlight the key aspects of each manuscript.

## Clinical studies

Intermittent fasting regimens have emerged as therapeutic tools to improve markers of non-alcoholic fatty liver disease and cardiovascular risk factors (9–15). Kord-Varkaneh, Salehi Sahlabadi et al. conducted a randomized controlled trial to assess the benefits of intermittent fasting 5:2 in patients with non-alcoholic fatty liver disease. The results of their research revealed that adherence to the 5:2 intermittent fasting regimen can decrease anthropometric indices (waist circumference, body weight, fat mass, and body mass index), several biochemical markers (alanine and aspartate aminotransferases, triglycerides, high-sensitivity C-reactive protein, and cytokeratin-18), as well as fibrosis and steatosis scores. Fasting plasma glucose, insulin, HOMA-IR, total cholesterol, low- and high-density lipoprotein cholesterol, as well as total antioxidant capacity levels, remained unaltered following the intervention (Kord-Varkaneh, Salehi Sahlabadi et al.).

Another evidence-based manuscript targeted the topic of non-alcoholic fatty liver disease. Kord-Varkaneh, Poursoleiman et al. performed a systematic review of the literature to assess the potential benefits of low-fat vs. low-carbohydrate diets in the aforementioned metabolic disorder and concluded that both dietary interventions are effective in reducing anthropometric indices and metabolic biomarkers, however, it appears that a reduced intake of fats were more likely to drop liver enzyme concentrations.

Li et al. conducted a systematic review and meta-analysis to assessed the impact of high-fructose corn syrup compared to sucrose on anthropometric indices and metabolic biomarkers. Their findings suggest that the two compounds are similar in terms of their effects on anthropometric indices and metabolic parameters, however, consumption of the former was linked to higher concentrations of C-reactive protein (a marker of systemic inflammation) (Li et al.).

Omega-3 polyunsaturated fatty acid supplementation (*n*-3 PUFAs) are essential components that can improve the glycemic and cardiovascular profiles in different populations (16, 17). Khorshidi et al. evaluated the effects of *n*-3 PUFA supplementation on adolescents diagnosed with type 1 diabetes mellitus through a randomized controlled trial. The researchers demonstrated that the intervention enhanced flow-mediated dilatation, and reduced triglyceride concentrations, without any notable impact on other biomarkers of endothelial or vascular function or on metabolic parameters (Khorshidi et al.).

Micronutrients, including vitamin D, are essential for health and against diseases Proper screening for vitamin D status is of global interest to prevent and possibly treat many ailments diseases (18–21). Employing a systematic review and meta-analysis approach, Fatahi et al. investigated the association between serum vitamin D concentrations and inflammatory bowel disease in children and adolescents. Their research indicated a tendency toward children and adolescents with inflammatory bowel disease to display vitamin D deficiency (Fatahi et al.).

Natural compounds are purported to modulate hormones to enhance men's heath (22–24). In their randomized controlled trial, Sadeghi et al. investigated the action of FruHis, a natural compound derived from the combination of fructose and histidine. This product was investigated alone or in combination with lycopene in the setting of benign prostatic hyperplasia. According to their findings, the co-administration of FruHis and lycopene reduced IGF-1 levels, without statistically significant changes in other clinical or laboratory parameters (Sadeghi et al.).

Herbal medicines can complement dietary approaches possibly due to their anti-inflammatory properties (25–28). Jiang et al. explored the impact of supplementation with echinacoside, a natural compound used in traditional Chinese medicine and extracted from *Herba Cistanches*, a type of medicinal herb, on immune parameters and key genes involved in the immune response in a murine model of exercise-induced injury. Their research highlighted its anti-inflammatory properties. In addition, they employed genetic studies and artificial intelligence techniques to propose a new method of screening for the detection of natural products that could elicit health benefits in humans by targeting relevant genes involved in immunity (Jiang et al.).

## Sport studies

Teixeira et al. investigated the effects of 8 weeks of protein supplementation with either a plant-based protein supplement or whey protein in high level futsal players. The futsal players were ingesting “sufficient” amounts of daily protein previously shown to optimize muscle performance (>1.6 g/kg/day) and therefore the supplements did provide any further benefit. These results are important for athletes and highlight that more protein is not always better and further corroborates that 1.6 g/kg/day of protein is optimal (Teixeira et al.).

In another longitudinal study, Cabre et al. randomized healthy active participants to either a pre-post multi-ingredient supplement or placebo in conjunction with a high intensity resistance training and high intensity interval training program. Overall, the supplement was able to positively enhance lean mass and muscular strength (both upper and lower body) in both men and women (Cabre et al.).

Rauch et al. conducted a systematic review with an aim to investigate the effects of various supplementation protocols of pre, pro, and syn-biotics in healthy active adults on gastrointestinal outcomes at rest and in response to acute exercise. Overall, 1,204 participants were included from 37 manuscripts. The review highlights that prebiotics can alter gut microbial composition and short-chain fatty acids (SCFA) concentrations, while probiotics increase the supplemented species-strain with limited effect on SCFA and no effects on gastrointestinal status markers at rest. Further, probiotics and synbiotic supplementation did not influence epithelial injury and permeability, systemic endotoxin and inflammation cytokine profiles, or gastro-intestinal symptoms in response to exercise (Rauch et al.).

Exercising in a hot and humid environment stresses the cardiovascular system and may impair performance. Roriz et al. performed a systematic review aimed to examine whether ice, cold beverages or menthol solutions can alter performance when exercising in different environmental conditions. Menthol solution appeared to improved physical performance during continuous endurance exercise in the heat. In contrast, ice ingestion or cold beverages did not consistently increase performance. Menthol with or within ice drinks resulted in a synergistic effect on performance. Interestingly, even in environmental conditions that are not extreme, internal cooling strategies may be ergogenic (Roriz et al.).

## Conclusion

This Special Issue included several high-quality original research articles and several rigorous systematic reviews that provided new insights for scientists and practitioners to help guide evidence based practice.

## Author contributions

All authors listed have made a substantial, direct, and intellectual contribution to the work and approved it for publication.
